# Associations between *GUCY1A3* genetic polymorphisms and large artery atherosclerotic stroke risk in Chinese Han population: a case-control study

**DOI:** 10.1186/s12944-019-1177-2

**Published:** 2019-12-28

**Authors:** Jian-li Li, Liu-yu Liu, Dong-dong Jiang, Yi-ying Jiang, Guo-qiu Zhou, Dong-can Mo, Man Luo

**Affiliations:** 1 Department of Neurology, First Affiliated Hospital of Guangxi Medical University, Nanning, 530021 China; 2 Guangxi Key Laboratory of Precision Medicine in Cardio-cerebrovascular Diseases Control and Prevention, Nanning, 530021 China; 3 Guangxi Clinical Research Center for Cardio-cerebrovascular Diseases, Nanning, 530021 China

**Keywords:** *GUCY1A3*, Polymorphism, Large artery atherosclerotic stroke, Atherosclerosis

## Abstract

**Background:**

Previous genome-wide association studies have found two single nucleotide polymorphisms (SNP) rs7692387 and rs1842896 located on or near the *GUCY1A3* gene were associated with coronary artery disease (CAD). *GUCY1A3* was considered to be involved in the process of atherosclerosis, but there was little information about the association between genotypic polymorphisms of the *GUCY1A3* and large artery atherosclerotic (LAA) stroke. This study aimed to investigate the associations between the *GUCY1A3* rs7692387, rs1842896 polymorphisms and LAA stroke susceptibility.

**Methods:**

A total of 298 LAA stroke patients and 300 control subjects from a southern Chinese Han population were included. SNaPshot technique was used for genotype analysis. Associations between genotypes and LAA stroke susceptibility were analyzed with logistic regression model.

**Results:**

Our study found that under the recessive model (TT vs. GT + GG), the *GUCY1A3* rs1842896 polymorphism was significantly correlated with LAA stroke (OR = 1.48, 95%CI: 1.07–2.04, *P* = 0.018). After adjustment for its effects on age, gender, cigarette smoking, total cholesterol, low-density lipoprotein cholesterol, HbA1c, hypertension, diabetes mellitus, and CAD, the rs1842896 TT genotype retained association with increased susceptibility to LAA stroke (recessive model: adjusted OR = 1.96, 95%CI: 1.22–3.17, *P* = 0.006). However, association between rs7692387 polymorphism with LAA stroke was not observed.

**Conclusion:**

Our results indicate that the GUCY1A3 rs1842896 polymorphism is an LAA stroke risk factor in Southern Han Chinese.

## Background

Ischemic stroke (IS) is a disease with high morbidity and mortality, which affects people across the world and causes heavy economic burden for families and the whole society [1]. According to the TOAST (Trial of Org 10,172 in Acute Stroke Treatment) classification [2], IS can be divided into five etiologic subtypes, including large artery atherosclerotic (LAA) stroke. LAA stroke carries the highest risk of early neurological deterioration and early recurrent stroke in comparison to other IS subtypes [3, 4]. LAA stroke is attributed to atherosclerosis, where the development of thrombosis may cause occlusion of cerebral artery, and lead to irreversible brain damage. Atherosclerosis is a typical complex disease process caused by multiple environmental and genetic factors and their interactions [5] . However, the causative genes are largely unknown. To date, genome-wide association studies (GWAS) have identified several genomic loci are associated with atherosclerosis-related diseases, such as LAA stroke and coronary artery disease (CAD). Some of these variants on chromosome 4q32.1 tags the *GUCY1A3* gene (according to a new nomenclature also known as *GUCY1A1*) [6, 7].

The *GUCY1A3* gene encodes the α1 subunit of the soluble guanylyl cyclase (sGC). The sGC complex, a heterodimeric enzyme comprising a β1 subunit and either an α1 or α2 subunit, is the only known receptor for nitric oxide (NO) and catalyzes the formation of the second messenger cyclic guanosine 3′,5′-monophosphate (cGMP). cGMP regulates a vast array of physiological processes, including neurotransmission [8], vascular smooth muscle cells (VSMCs) relaxation [9], and inhibition of platelet aggregation [10]. Previous GWAS have found rs7692387 [11] and rs1842896 [6] single nucleotide polymorphisms (SNPs) located on or near the *GUCY1A3* gene were associated with coronary artery disease (CAD). Although the nidi of CAD and LAA stroke are different, they share the same pathophysiological basis: atherosclerosis.

*GUCY1A3* was considered to be involved in the process of atherosclerosis, but there was little information about the association between genotypic polymorphisms of the *GUCY1A3* and large artery atherosclerotic (LAA) stroke. To our knowledge, the genetic evidence on the associations between the *GUCY1A3* rs7692387, rs1842896 polymorphisms and susceptibility of LAA stroke has not been reported previously. Therefore, the present study was conducted to evaluate the associations between the two SNPs and LAA stroke susceptibility in a Southern Chinese Han population.

## Methods

### Study population

A total of consecutive unrelated 298 LAA stroke patients were enrolled from September 2016 to December 2017 in this study. IS was defined according to the World Health Organization definition as “rapidly developing clinical signs of focal (or global) disturbance of cerebral function lasting more than 24 hours with no apparent cause other than of vascular origin”, with evidence of cerebral infarction in clinically relevant areas of the brain confirmed by computed tomography (CT) and/or magnetic resonance imaging (MRI). LAA stroke was diagnosed by experienced neurologists according to the TOAST classification [2]. Patients were enrolled if they: 1) aged 18 years or older; 2) were Southern Han Chinese; 3) had available blood samples. The following exclusion criteria were applied: the presence of other types of cerebrovascular disease (e.g. intracranial hemorrhage, subarachnoid hemorrhage, transient ischemic attack, cerebral aneurysm or cerebrovascular malformation), known embolic source (aortic arch, cardiac or carotid), severe systemic diseases such as neoplasms, autoimmune or inflammatory diseases, and serious chronic diseases (e.g. hepatic or renal failure). Three hundred symptom-free individuals were recruited as the control group. The inclusion criteria of control subjects were as follows: 1) aged 18 years or older; 2) were Southern Han Chinese; 3) had no history of cerebrovascular diseases; 4) had no history of atherosclerotic diseases; 5) had regular physical examinations. Exclusion criteria were the same as the criteria used for the patient group. The study was approved by the ethics committee of the First Affiliated Hospital of Guangxi Medical University and was carried out in accordance with the Declaration of Helsinki. All participants provided informed consent. All methods were performed in accordance with the relevant guidelines and regulations.

### Genotyping

Genomic DNA was isolated from a 300 μL blood sample using the TIANamp Blood DNA Kit (Tiangen Biotect [Beijing] Co., China) according to the manufacturer’s instructions. Extracted genomic DNA samples were stored at − 80 °C until genotyping was performed. Two SNPs in *GUCY1A3* gene, rs1842896 and rs7692387, were genotyped using the method of SNaPshot kit commercialized by Applied Biosystems, as described previously [12]. The sequences of primers used for polymerase chain reaction (PCR) and minisequencing extension were listed in Table 1. Fluorescent-labeled PCR fragments were resolved by capillary electrophoresis on an ABI PRISM 3130xl Genetic Analyzer (Applied Biosystems, Foster City, CA, USA). The data produced were analyzed with GeneMapper 4.1 software (Applied Biosystems).

**Table 1:** Primer sequences for *GUCY1A3* SNaPshot genotyping

Primer description	Sequence
rs1842896 PCR primer	Forward: GAATTATACAGTTACTTGCCTCAGGTTGAC
	Reverse: GAAAAGGCAGATTAAGATGACGGATTT
rs7692387 PCR primer	Forward: CACTTCACTGTGGAAACCGAGTTG
	Reverse: CAAAGCAAGTAAGGCAGAGAACAAGA
rs1842896 SR1 primer	CATGATAACTAGTGCTTTTAAGGGAATG
rs1842896 SR2 primer	TTTTTTTTTTTTCATGATAACTAGTGCTTTTAAGGGAATG
rs1842896 SR3 primer	TTTTTTTTTTTTTTTTTTTTTTTTCATGATAACTAGTGCTTTTAA-GGGAATG
rs7692387 SF1 primer	GGCCAAGGGCAGAGACATTT
rs7692387 SF2 primer	TTTTTTTTTTTTGGCCAAGGGCAGAGACATTT
rs7692387 SF3 primer	TTTTTTTTTTTTTTTTTTTTTTTTGGCCAAGGGCAGAGACATTT

### Statistical analysis

SPSS 22.0 software (IBM Inc., Chicago, IL, USA) was used for the statistical analysis. Alleles were tested for deviation from Hardy-Weinberg equilibrium (HWE) using a chi-square test. Continuous variables were expressed as mean ± SD. Normality of the sample distribution of each continuous variable was tested with the Kolmogorov-Smirnov test. Differences of continuous variables were evaluated by the student’s t-test or Mann-Whitney U test. Categorical variables were expressed as proportions and compared using a chi-square test. The genetic association was estimated using a univariate logistic regression analysis. A multivariate logistic regression analysis was performed to adjust for potential confounding factors, such as age, gender, cigarette smoking, total cholesterol, low-density lipoprotein cholesterol, Hemoglobin A1c (HbA1c), hypertension history, diabetes mellitus history, and coronary artery disease history. All tests were two-tailed and a *P*-value of 0.05 was considered statistically significant.

## Results

### Clinical characteristics of the study population

The clinical characteristics of LAA stroke patients and control subjects are summarized in Table 2. A total of 298 patients with LAA stroke, 206 (69.13%) males and 92 (30.87%) females, were enrolled in the case group. A total of 300 volunteers, 194 (64.67%) males and 106 (35.33%) females, were enrolled in the control group. The mean age was 67.58 ± 12.62 years in the case group and 63.29 ± 11.80 years in the control group. As shown in Table 2, the prevalence of conventional risk factors such as hypertension, diabetes mellitus, and cigarette smoking, was found to be higher in patients compared to the controls. Patients with LAA stroke had a higher prevalence of coronary artery disease history (8.05 vs. 3.67%) than controls. The level of HbA1c was significantly higher in LAA stroke patients when compared to controls.

**Table 2:** Clinical characteristics of cases and controls

	Cases (*n* = 298)	Controls (*n* = 300)
Age, years	67.58 ± 12.62*	63.29 ± 11.80
Male, n (%)	206 (69.13%)	194 (64.67%)
BMI, kg/m^2^	23.72 ± 3.53	23.32 ± 3.22
SBP, mmHg	148.39 ± 21.90*	128.87 ± 19.99
DBP, mmHg	85.58 ± 14.68*	77.78 ± 13.14
Total Cholesterol, mmol/L	4.93 ± 1.28	4.88 ± 1.28
Triglyceride, mmol/L	1.51 ± 0.91	1.49 ± 0.93
Low-Density Lipoprotein Cholesterol, mmol/L	3.29 ± 1.09	3.21 ± 1.06
Hemoglobin A1c, %	6.69 ± 1.53*	6.24 ± 1.24
Cigarette smoking, n (%)	110 (36.91%)*	71 (23.67%)
Coronary artery disease history, n (%)	24 (8.05%)*	11 (3.67%)
Hypertension history, n (%)	198 (66.44%)*	98 (32.67%)
Diabetes mellitus history, n (%)	65 (21.81%)*	30 (10.00%)

Age, body mass index (BMI), systolic blood pressure (SBP), diastolic blood pressure (DBP), total cholesterol, triglyceride, low-density lipoprotein cholesterol, glucose and Hemoglobin A1c (HbA1c) values are shown as the mean (standard deviation), and other values as the number of individuals (n) with percentage (n/total N) in parentheses.

*P < 0.05 vs. control

### Genotype distributions of the *GUCY1A3* SNPs

The genotype distribution of the *GUCY1A3* rs1842896 and rs7692387 in LAA stroke patients and controls are given in Table 3. The distribution of rs1842896 genotypes in LAA stroke patients significantly differed from the control group (*P* = 0.028), while the distribution of rs7692387 genotypes was similar between case and control individuals (*P* = 0.915). The TT, GT and GG genotype frequencies of rs1842896 were 54.36, 36.58 and 9.06% in patients, 44.67, 47.33, and 8.00% in controls, respectively. Obtained genotype frequencies for each SNP did not significantly deviate from Hardy-Weinberg equilibrium expectations (*P* > 0.05).

**Table 3:** Distributions of the *GUCY1A3* rs1842896 and rs7692387 genotypes

Genotypes	Cases (*n* = 298)	Controls (*n* = 300)	*χ2*-value	*P*-value
rs1842896				
TT (%)	162 (54.36%)	134 (44.67%)	7.157	0.028*
GT (%)	109 (36.58%)	142 (47.33%)		
GG (%)	27 (9.06%)	24 (8.00%)		
rs7692387				
GG (%)	184 (61.74%)	190 (63.33%)	0.178	0.915
GA (%)	102 (34.23%)	99 (33.00%)		
AA (%)	12 (4.03%)	11 (3.67%)		

*P*-values are based on chi-square test for genotype comparison

The entries in boldface are statistically significant

*P < 0.05

### Associations between the *GUCY1A3* SNPs and LAA stroke risk

We assessed the associations of the *GUCY1A3* rs1842896 and rs7692387 polymorphisms with LAA stroke in different genetic models, including dominant, recessive, and additive models. Genotypic association between rs1842896 and LAA stroke was significant in the recessive model (recessive model: OR = 1.48, 95% CI: 1.07–2.04, *P* = 0.018, Table 4), there was no statistical significance in the other models (Table 4). After adjusted for age, gender, cigarette smoking, total cholesterol, low-density lipoprotein cholesterol, HbA1c, hypertension history, diabetes mellitus history, and coronary artery disease history, TT genotype of rs1842896 was still associated with an increased risk of LAA stroke in the recessive model (adjusted OR = 1.96, 95% CI: 1.22–3.17, *P* = 0.006, Table 4). However, the rs7692387 polymorphism failed to show significant association in all three genetic models.

**Table 4:** Associations between the *GUCY1A3* rs1842896 and rs7692387 with LAA stroke

Model	Crude OR (95% CI)	*P*-value	Adjusted OR (95% CI)	Adjusted *P*-value
rs1842896				
Dominant (TT + GT vs. GG)	0.87 (0.49–1.55)	0.643	0.79 (0.34–1.83)	0.586
Recessive (TT vs. GT + GG)	1.48 (1.07–2.04)	0.018*	1.96 (1.22–3.17)	0.006*
Additive (TT vs. GG)	1.08 (0.59–1.95)	0.813	1.22 (0.51–2.93)	0.656
rs7692387				
Dominant (GG + GA vs. AA)	0.91 (0.39–2.09)	0.819	1.48 (0.51–4.33)	0.472
Recessive (GG vs. GA + AA)	0.93 (0.67–1.30)	0.688	0.71 (0.43–1.17)	0.177
Additive (GG vs. AA)	0.89 (0.38–2.06)	0.782	1.28 (0.43–3.75)	0.657

Adjusted for age, gender, cigarette smoking, total cholesterol, low-density lipoprotein cholesterol, HbA1c, hypertension history, diabetes mellitus history, and coronary artery disease history

The entries in boldface are statistically significant

*P < 0.05

## Discussion

We designed this study to investigate the associations between the *GUCY1A3* polymorphisms and LAA stroke in Chinese Han population. Our results showed that, under the recessive model, the *GUCY1A3* rs1842896 TT genotype was significantly correlated with LAA stroke. After adjustment for its effects on age, gender, cigarette smoking, total cholesterol, low-density lipoprotein cholesterol, HbA1c, hypertension, diabetes mellitus, and CAD, the rs1842896 TT genotype retained association with increased susceptibility to LAA stroke (recessive model: adjusted OR = 1.96, 95%CI: 1.22–3.17, *P* = 0.006), indicating the SNP might be associated with LAA stroke independently of its effects on hypertension, diabetes mellitus and CAD. This association suggested that the risk for LAA stroke in the rs1842896 TT genotype carriers were increased 1.96-fold when compared with GG/GT genotype carriers. However, association between the rs7692387 polymorphism with LAA stroke was not observed.

The rs1842896 is located 76.4 kb upstream of the *GUCY1A3* locus. The *GUCY1A3* gene encodes the α1 subunit of sGC, the major cellular receptor for NO that is implicated in pathogenesis of atherosclerosis. sGC acts as a heterodimer composed of an α subunit and a β subunit. Although two isoforms of each subunit (α1, α2, β1 and β2) exist, only the α1β1 heterodimer and the α2β1 heterodimer are functional enzymes [13]. The α1β1 heterodimer is the most abundant isoform distributed ubiquitously and displays higher activity than α2β1, so it is regarded as canonical sGC [14]. As a key enzyme of the NO signaling pathway, sGC can change the conversion of GTP into cGMP, leading to increased production of cGMP. Increasing intracellular levels of cGMP and the subsequent intracellular processes lead to relaxation of VSMCs and inhibition of platelet aggregation [14, 15], preventing the formation of atherosclerosis. Previous studies have explored the therapeutic potential of sGC stimulators [16].

The impact of genetic variation in the *GUCY1A3* gene on human atherosclerosis has been highlighted by previous studies. In 2012, a meta-analysis of two GWAS in Chinese Han population with 1515 CAD cases and 5019 controls identified rs1842896 at 4q32.1 near *GUCY1A3* showed significant association with CAD [6]. Later, the CARDIoGRAMplusC4D Consortium [11] also detected a strong association between a variant at the *GUCY1A3* locus and CAD. Recently, an extended meta-analysis [7] which considerably expanded the coverage of human genetic variation especially for low-frequency variants to determine whether these variants mediate risk for IS found that *GUCY1A3* showed suggestive association with LAA stroke. Besides, it has been shown that rare coding variants in the *GUCY1A3* gene were linked in families to myocardial infarction [17] and stroke [18] risk.

The risk variants in the *GUCY1A3* gene leading to reduced sGC availability and activity may be one reason of increased risk in atherosclerotic events. Kessler et al. found *GUCY1A3* gene variant was associated with both reduced platelet α1 sGC protein levels and increased atherosclerotic disease burden [19]. It has also been shown that absence or reduction of α1 subunit protein was detectable in variants in the *GUCY1A3* gene identified in sporadic young myocardial infarction cases [20]. There are several limitations of our study that have to be taken into account. First, our study population was limited to southern Chinese individuals. As genetic influence is strongly relevant to ethnic background, our findings need to be validated in other ethnic populations. Second, our study sample size was relatively small. Third, biological functions were not conducted which were deserved further research. Fourth, it has been demonstrated that nutraceuticals and functional food ingredients can reduce the IS risk [21, 22]. However, we were not able to collect the important risk factor for subjects who participated in the study. Further studies solving these limitations are needed.

## Conclusion

To our knowledge, this is the first study to demonstrate an association between the GUCY1A3 rs1842896 and LAA stroke. We found that the *GUCY1A3* rs1842896 TT genotype was associated with an increased susceptibility to LAA stroke in a Chinese Han population. This finding indicates a potential link between *GUCY1A3* and risk of LAA stroke. Further research will be required to explore the role of *GUCY1A3* in the pathogenesis of LAA stroke.

## Data Availability

The datasets supporting the conclusions of this article are included within the article.

## Abbreviations

BMI: Body mass index
CAD: Coronary artery disease
cGMP: cyclic guanosine 3′,5′-monophosphate
DBP: Diastolic blood pressure
GWAS: Genome-wide association studies
HbA1C: Hemoglobin A1c
HWE: Hardy-Weinberg equilibrium
IS: Ischemic stroke
LAA: Large artery atherosclerotic
MRI: Magnetic resonance imaging
NO: Nitric oxide
PCR: Polymerase chain reaction
SBP: Systolic blood pressure
sGC: Soluble guanylyl cyclase
SNP: Single nucleotide polymorphisms
TOAST: Trial of Org 10,172 in Acute Stroke Treatment
VSMCs: Vascular smooth muscle cells

## References

[1] Krishnamurthi RV, Feigin VL, Forouzanfar MH, Mensah GA, Connor M, Bennett DA, Moran AE, Sacco RL, Anderson LM, Truelsen T (2013). Global and regional burden of first-ever ischaemic and haemorrhagic stroke during 1990-2010: findings from the global burden of disease study 2010. Lancet Glob Health.

[2] Adams HJ, Bendixen BH, Kappelle LJ, Biller J, Love BB, Gordon DL, Marsh ER (1993). Classification of subtype of acute ischemic stroke. Definitions for use in a multicenter clinical trial. TOAST. Trial of org 10172 in acute stroke treatment. STROKE.

[3] Lovett JK, Coull AJ, Rothwell PM (2004). Early risk of recurrence by subtype of ischemic stroke in population-based incidence studies. NEUROLOGY..

[4] Larry BG, Tsivgoulis G, Katsanos AH, Alexandrov AV (2016). Statin pretreatment is associated with better outcomes in large artery atherosclerotic stroke. Neurology.

[5] Li D, Budoff MJ (2016). Genetics paired with CT angiography in the setting of atherosclerosis. Clin Imaging.

[6] Lu X, Wang L, Chen S, He L, Yang X, Shi Y, Cheng J, Zhang L, Gu CC, Huang J (2012). Genome-wide association study in Han Chinese identifies four new susceptibility loci for coronary artery disease. Nat Genet.

[7] Malik R, Traylor M, Pulit SL, Bevan S, Hopewell JC, Holliday EG, Zhao W, Abrantes P, Amouyel P, Attia JR (2016). Low-frequency and common genetic variation in ischemic stroke: the METASTROKE collaboration. Neurology.

[8] Luo C, Gangadharan V, Bali KK, Xie RG, Agarwal N, Kurejova M, Tappe-Theodor A, Tegeder I, Feil S, Lewin G (2012). Presynaptically localized cyclic GMP-dependent protein kinase 1 is a key determinant of spinal synaptic potentiation and pain hypersensitivity. PLoS Biol.

[9] Groneberg D, Konig P, Wirth A, Offermanns S, Koesling D, Friebe A (2010). Smooth muscle-specific deletion of nitric oxide-sensitive guanylyl cyclase is sufficient to induce hypertension in mice. Circulation.

[10] Wobst J, Kessler T, Dang TA, Erdmann J, Schunkert H (2015). Role of sGC-dependent NO signalling and myocardial infarction risk. J Mol Med (Berl).

[11] Deloukas P, Kanoni S, Willenborg C, Farrall M, Assimes TL, Thompson JR, Ingelsson E, Saleheen D, Erdmann J, Goldstein BA (2013). Large-scale association analysis identifies new risk loci for coronary artery disease. Nat Genet.

[12] Li W, Hu B, Li GL, Zhao XQ, Xin BZ, Lin JX, Shen Y, Liang XH, Liu GF, Gao HQ (2012). Heterozygote genotypes at rs2222823 and rs2811712 SNP loci are associated with cerebral small vessel disease in Han Chinese population. CNS Neurosci Ther.

[13] Rothkegel C, Schmidt PM, Atkins DJ, Hoffmann LS, Schmidt HH, Schroder H, Stasch JP (2007). Dimerization region of soluble guanylate cyclase characterized by bimolecular fluorescence complementation in vivo. Mol Pharmacol.

[14] Wobst J, Schunkert H, Kessler T (2018). Genetic alterations in the NO-cGMP pathway and cardiovascular risk. Nitric Oxide.

[15] Wallace S, Guo DC, Regalado E, Mellor-Crummey L, Bamshad M, Nickerson DA, Dauser R, Hanchard N, Marom R, Martin E (2016). Disrupted nitric oxide signaling due to GUCY1A3 mutations increases risk for moyamoya disease, achalasia and hypertension. Clin Genet.

[16] Stasch JP, Pacher P, Evgenov OV (2011). Soluble guanylate cyclase as an emerging therapeutic target in cardiopulmonary disease. Circulation.

[17] Erdmann J, Stark K, Esslinger UB, Rumpf PM, Koesling D, de Wit C, Kaiser FJ, Braunholz D, Medack A, Fischer M (2013). Dysfunctional nitric oxide signalling increases risk of myocardial infarction. Nature.

[18] Hervé D, Philippi A, Belbouab R, Zerah M, Chabrier S, Collardeau-Frachon S, Bergametti F, Essongue A, Berrou E, Krivosic V (2014). Loss of α1β1 soluble guanylate cyclase, the major nitric oxide receptor, leads to moyamoya and achalasia. Am J Hum Genet.

[19] Kessler T, Wobst J, Wolf B, Eckhold J, Vilne B, Hollstein R, von Ameln S, Dang TA, Sager HB, Moritz RP (2017). Functional characterization of the GUCY1A3 coronary artery disease risk locus. Circulation.

[20] Wobst J, von Ameln S, Wolf B, Wierer M, Dang TA, Sager HB, Tennstedt S, Hengstenberg C, Koesling D, Friebe A (2016). Stimulators of the soluble guanylyl cyclase: promising functional insights from rare coding atherosclerosis-related GUCY1A3 variants. Basic Res Cardiol.

[21] Scicchitano P, Cameli M, Maiello M, Modesti PA, Muiesan ML, Novo S, Palmiero P, Saba PS, Pedrinelli R, Ciccone MM (2014). Nutraceuticals and dyslipidaemia: beyond the common therapeutics. J Funct Foods.

[22] Boehme AK, Esenwa C, Elkind MS (2017). Stroke risk factors, genetics, and prevention. Circ Res.

